# A nomogram to predict survival in non-small cell lung cancer patients treated with nivolumab

**DOI:** 10.1186/s12967-019-1847-x

**Published:** 2019-03-27

**Authors:** Andrea Botticelli, Massimiliano Salati, Francesca Romana Di Pietro, Lidia Strigari, Bruna Cerbelli, Ilaria Grazia Zizzari, Raffaele Giusti, Marco Mazzotta, Federica Mazzuca, Michela Roberto, Patrizia Vici, Laura Pizzuti, Marianna Nuti, Paolo Marchetti

**Affiliations:** 1 Department of Clinical and Molecular Medicine, Sant’Andrea Hospital, Sapienza University of Rome, Rome, Italy; 2 Department of Oncology, University Hospital of Modena and Reggio Emilia, Modena, Italy; 3 Division of Molecular Pathology, The Institute of Cancer Research and Gastrointestinal Unit, The Royal Marsden Hospital, London, Sutton, UK; 4 Laboratory of Medical Physics and Expert Systems, Regina Elena National Cancer Institute, Rome, Italy; 5 Department of Radiological, Oncological and Pathological Sciences, Sapienza University of Rome, Rome, Italy; 6 Department of Experimental Medicine, Sapienza University of Rome, Rome, Italy; 7 Division of Medical Oncology 2, IRCCS Regina Elena National Cancer Institute, Rome, Italy

**Keywords:** Immunotherapy, Lung cancer, Prognostic factors, Nivolumab, Nomogram

## Abstract

**Background:**

The advent of immune checkpoint inhibitors (ICIs) has considerably expanded the armamentarium against non-small cell lung cancer (NSCLC) contributing to reshaping treatment paradigms in the advanced disease setting. While promising tissue- and plasma-based biomarkers are under investigation, no reliable predictive factor is currently available to aid in treatment selection.

**Methods:**

Patients with stage IIIB–IV NSCLC receiving nivolumab at Sant’Andrea Hospital and Regina Elena National Cancer Institute from June 2016 to July 2017 were enrolled onto this study. Major clinicopathological parameters were retrieved and correlated with patients’ survival outcomes in order to assess their prognostic value and build a useful tool to assist in the decision making process.

**Results:**

A total of 102 patients were included in this study. The median age was 69 years (range 44–85 years), 69 (68%) were male and 52% had ECOG PS 0. Loco-regional/distant lymph nodes were the most commonly involved site of metastasis (71%), followed by lung parenchyma (67%) and bone (26%). Overall survival (OS) in the whole patients’ population was 83.6%, 63.2% and 46.9% at 3, 6 and 12 months, respectively; while progression-free survival (PFS) was 66.5%, 44.4% and 26.4% at 3, 6 and 12 months, respectively. At univariate analysis, age ≥ 69 years (*P *= 0.057), ECOG PS (*P *< 0.001), the presence of liver (*P *< 0.001), lung (*P *= 0.017) metastases, lymph nodes only involvement (P = 0.0145) were significantly associated with OS and ECOG PS (*P *< 0.001) and liver metastases (*P *< 0.001), retained statistical significance at multivariate analysis. A prognostic nomogram based on three variables (liver and lung metastases and ECOG PS) was built to assign survival probability at 3, 6, and 12 months after nivolumab treatment commencement.

**Conclusion:**

We developed a nomogram based on easily available and inexpensive clinical factors showing a good performance in predicting individual OS probability among NSCLC patients treated with nivolumab. This prognostic device could be valuable to clinicians in more accurately driving treatment decision in daily practice as well as enrollment onto clinical trials.

## Background

Lung cancer represents a massive health burden worldwide with 1.7 million deaths annually and a 26% increase in incidence during the last decade [1]. More than a half of patients present with stage IV disease and less than 5% of them survive beyond 5 years [2].

The introduction of immune checkpoint inhibitors (ICI) has considerably expanded the armamentarium against non-small cell lung cancer (NSCLC) contributing to reshaping treatment paradigms in the advanced disease setting [3, 4]. The anti-PD-1 pembrolizumab both as monotherapy and combined with platinum/pemetrexed doublet is considered a first-line treatment option in PD-L1 overexpressing (≥ 50%) [5] and unselected patients [6], respectively, in absence of actionable oncogenic drivers. Moreover, the anti-PD-L1 atezolizumab has emerged as a further front-line therapeutic choice both in combination with bevacizumab, carboplatin and paclitaxel [7] and platinum-based doublets [8] in NSCLC regardless of PD-L1 status. In the second-line setting, nivolumab [9] and atezolizumab [10] (irrespective of PD-L1 expression) and pembrolizumab (PD-L1 ≥ 1%) [11] are approved as single-agent for chemotherapy pretreated, immunotherapy-naïve patients.

The fast-growing number of immunotherapeutics and their limited efficacy with 70–80% of patients progressing within the first 2–3 months underline the need for predictive biomarkers aiding in treatment selection [12]. Moreover, a subset of patients termed as hyperprogressors and ranging from 9 to 29% have been described that experience a paradoxically accelerated tumour growth while on ICI treatment [13]. Tumour-associated macrophages reprogramming towards a pro-tumorigenic phenotype upon Fc receptor engagement by ICI has been suggested to have a causative role in this phenomenon in patients with distinctive immune and genetic profiles.

Nivolumab is a fully human IgG4 anti-PD1 monoclonal antibody that showed to prolong OS compared to docetaxel in NSCLC failing first-line chemotherapy. However, it yielded a response rate as low as 13.6% to 23% and a median PFS of 2.3 to 4 months in biomarker-unselected patients [9, 10]. Several biomarkers are being studied that can help to enrich for patients more likely to benefit from nivolumab [14, 15]. PD-L1 is a suboptimal predictive biomarker since less than 50% of PD-L1-selected patients respond to treatment and some responders may be encountered also in ‘biomarker-negative’ cohorts. Tumour mutational burden (TMB) holds great promise and up to now is the sole clinically validated biomarker. Nevertheless, no consensus exists on how it should be measured and its widespread use is thus limited. Additional promising tissue- and plasma-based predictive biomarkers are under investigation, including tumour infiltrating lymphocytes, “immunoscore” (composite biomarker integrating four T cell related IHC features), immune gene signatures, eosinophil, lymphocyte and neutrophil counts and relative ratios from peripheral blood, plasma IL-6 and IDO, microsatellite instability status, interferon signature, T cell repertoire, MHC status and microbiome profile [14]. Among clinical factors, poor performance status (ECOG PS ≥ 2), a period of time since prior treatment ≥ 6 months and involvement of more than one metastatic site have been independently associated with shorter OS in a cohort of 175 pretreated NSCLC patients receiving nivolumab [16]. More recently, ECOG PS ≥ 2, liver and lung metastases have been suggested to be independent predictors of nivolumab efficacy in an Asian population of 201 advanced NSCLC [17].

The aim of our study is to assess the predictive-prognostic significance of clinicopathological parameters in NSCLC patients receiving second-line nivolumab treatment in clinical practice in order to build a useful tool to assist in the decision making process.

## Materials and methods

### Patients

Patients with stage IIIB-IV NSCLC receiving nivolumab at Sant’Andrea Hospital and Regina Elena National Cancer Institute from June 2016 to July 2017 were enrolled onto this study. Inclusion criteria were: age > 18 years; histologically-documented diagnosis of NSCLC; Eastern Cooperative Oncology Group (ECOG) performance status ≤ 2; measurable disease; progression on or after first-line platinum-containing doublet; patients harboring oncogenic driver aberrations (i.e. EGFR mutations or ALK fusion oncogene) were required to have received previous tyrosine kinase inhibitor therapy; adequate cardiac, pulmonary, renal, liver and bone marrow function; patients with stable and asymptomatic central nervous system metastases were eligible. Exclusion criteria were: autoimmune disease; symptomatic interstitial lung disease and any other significant comorbidity; systemic immunosuppression; prior treatment with immune-stimulatory antitumor agents including checkpoint-targeted agents. All patients gave written informed consent.

The study was conducted in accordance with good clinical practice guidelines and the declaration of Helsinki. The final version of the protocol was approved by the Institutional Ethics Committee of the two Institutions involved.

### Treatment, efficacy and safety assessments

Nivolumab was administered intravenously at a standard dose of 3 mg/kg every 2 weeks until disease progression or development of unacceptable toxicity. Tumour response was assessed at week 9 and every 6 weeks thereafter until disease progression using immune-related Response Evaluation Criteria in Solid Tumors Criteria (i-RECIST) and classified according to disease control (complete response, partial response and stable disease) and progressive disease. Safety assessments were performed at day 1 of every cycle until the end of treatment and toxicities were graded according to the National Cancer Institute Common Terminology Criteria for Adverse Events (version 4.0).

### Objectives and outcomes

Progression-free survival (PFS) was defined as the time from nivolumab commencement until the first documented tumour progression or death from any cause, whichever occurred first. Overall survival (OS) was defined as the time from nivolumab commencement to death from any cause.

Early progressors patients were defined as those experiencing disease progression within 3 months from the beginning of nivolumab treatment.

The association between early progression and clinicopathological factor PS, age, sex and site of metastases was assessed together with the association between PFS > 12 months and PS, age, sex and site of metastases.

### Statistical analysis

Categorical variables are presented as a number with a percentage in descriptive tables, and they were compared with Fisher’s exact test or Pearson’s Chi-square test. The impact of clinicopathological variables on overall survival (OS) and progression-free survival (PFS) was analyzed by both the univariate and multivariate analyses (UVA and MVA, respectively). With regards to UVA, patients’ OS and PFS were analyzed using the Kaplan–Meier method and log-rank tests. Prognostic clinic-pathological variables deemed of potential relevance in the univariate analysis (corresponding to a cutoff of P < 0.10) were included in the multivariate Cox proportional hazards regression analysis. A nomogram to predict 3, 6, and 12-months survival probability was developed based on covariates retaining a statistically significant power (*P *< 0.05) in MVA. To quantify the discrimination performance of the nomogram, Harrell’s C-index was measured. The nomogram was subjected to bootstrapping validation (1000 bootstrap resamples) to calculate a relatively corrected C-index. Calibration was studied graphically after grouping patients into deciles with respect to their predicted probabilities and plotting the mean predicted probabilities against the mean observed probabilities. Bootstrapping was applied to correct the model based on the estimated optimism. Internal validation was performed determining the OS in each calculated group. Discrimination of nomogram was tested by Kaplan–Meier curves and boxplots. A *P *< 0.05 was considered statistically significant. Statistical analyses were performed using R-package software.

## Results

### Patients

A total of 102 patients fulfilled the inclusion criteria and were enrolled in this study. Overall, the median age was 69 years (range 44–85 years), Sixty-nine (68%) were male and 52% had ECOG PS 0. Loco-regional/distant lymph nodes were the most commonly involved site of metastasis (71%), followed by lung parenchyma (67%) and bone (26%). Other baseline clinicopathological parameters are reported in Table 1. Seventy-two (88%) patients experienced early progression, while seven (39%) patients presented PFS longer than 12 months.

**Table 1:** Baseline clinico-pathological characteristics (n = 102)

Parameter	N (%)
Age years (median, range)	69 (44–85)
Height	168 cm (152–186)
Weight	69.5 kg (45–175)
Gender	
Male	69 (68%)
Female	33 (32%)
ECOG PS	
0	53 (52%)
1	41 (40%)
2	8 (8%)
Lung parenchyma metastasis	
Yes	68 (67%)
No	34 (34%)
Lymph node metastasis	
Yes	72 (71%)
No	30 (29%)
Loco-regional lymph node metastasis	
Yes	31 (30%)
No	71 (70%)
Liver metastasis	
Yes	19 (19%)
No	83 (81%)
Brain metastasis	
Yes	14 (14%)
No	88 (86%)
Malignant pleural effusion	
Yes	9 (9%)
No	93 (91%)
Bone metastasis	
Yes	27 (26%)
No	75 (74%)
Adrenal gland metastasis	
Yes	11 (11%)
No	91 (89%)
N. of metastatic sites	
1	21 (21%)
2	34 (33%)
3	47 (46%)

After a median follow-up period of 11 months (range 1–29 months), all 102 treated patients were assessable for OS. At the time of the analysis, 55 (54%) patients had died. OS in the whole patients’ population was 83.6%, 63.2% and 46.9% at 3, 6 and 12 months, respectively; while PFS was 66.5%, 44.4% and 26.4% at 3,6 and 12 months, respectively.

### Prognostic factors, nomogram development and performance

**Fig. 1:**
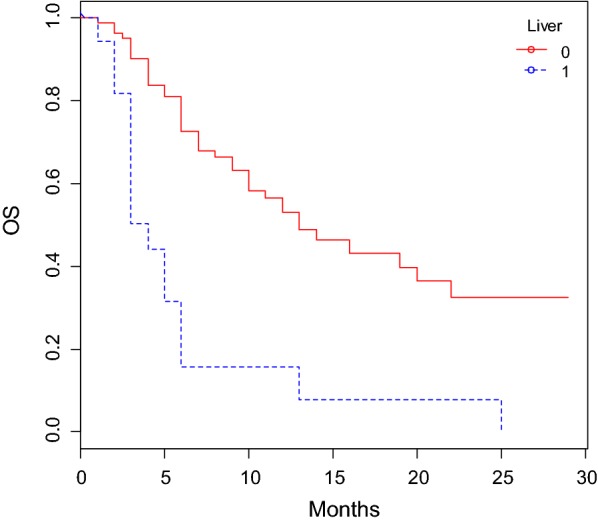
OS according to liver metastases status

**Fig. 2:**
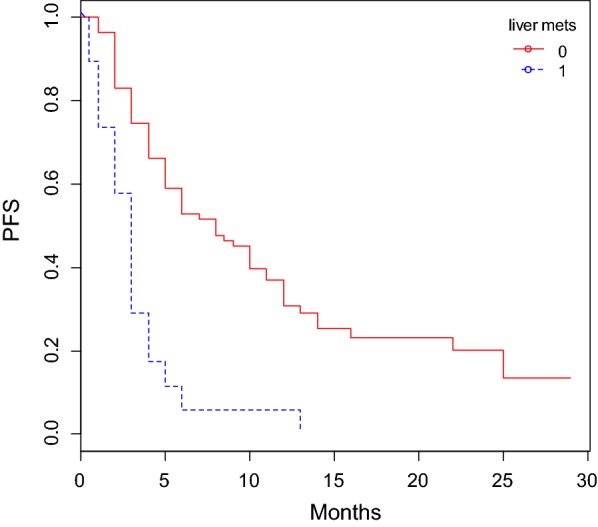
PFS according to liver metastases status

The OS was significantly shorter in patients aged > 69 years old compared to those younger than 69 years old. Patients with liver metastases (Figs. 1, 2) experienced a significantly lower OS, while those affected by lung metastatic deposits lived longer.

**Fig. 3:**
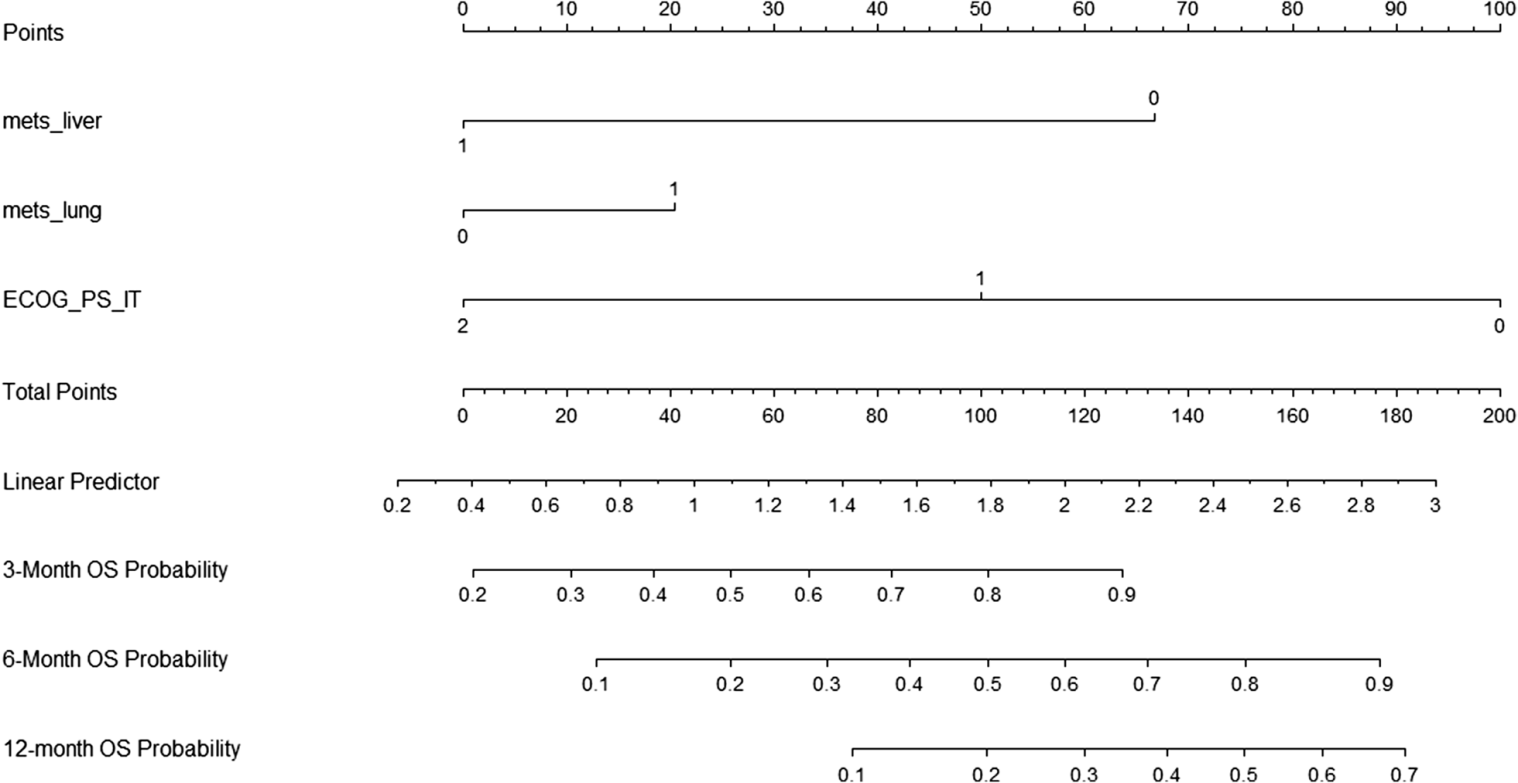
Prognostic nomogram for NSCLC patients to assign their probability of survival at 3, 6, and 12-months after nivolumab treatment initiation. The probability of survival at 3, 6, and 12 months can be obtained as function of total points calculated as the sum of points for each specific variable. Points are assigned for each risk factor by drawing a line upward from the corresponding values to the ‘point’ line. The total sum of points for three risk factors is plotted on the ‘total points’ line. A line is drawn down to read the corresponding predictions of 3-, 6-, 12-month-survival probability

At UVA assuming the cutoff of P < 0.10 for include potential interest parameters, age ≥ 69 years (*P *= 0.057), ECOG PS (*P *< 0.001), the presence of liver (*P *< 0.001), lung (*P *= 0.017) metastases, lymph nodes only involvement (*P *= 0.0145) were significantly associated with OS and were included in the multivariate analysis. ECOG PS (*P *< 0.001) and liver metastases (*P *< 0.001), retained statistical significance at MVA (Tables 2, 3). Based on the estimated regression coefficients in the Cox analysis, a prognostic nomogram that included liver, lung metastases and ECOG PS was developed to assign survival probability at 3, 6, and 12 months after nivolumab treatment commencement (Fig. 3).

**Table 2:** Univariate and multivariate analysis for OS

Covariate	Univariate analysis		Multivariate analysis	
	HR (95% CI)	P value	HR (95% CI)	P value
Age ≥ 69 years	*0.6389 (0.3691–1.1060)*	*0.057*	–	–
Sex	1.4 (0.7892**–**2.485)	0.247	–	–
Weight ≥ 69.5 kg	1.1999 (0.6606**–**2.1794)	0.543	–	–
Height > 168 cm	0.9492 (0.4884**–**1.8445)	0.876	–	–
Liver metastasis	*3.6912 (2.0123–6.7710)*	*0.000*	*3.0637 (1.6693–.623)*	*0.0003*
Lung metastasis	*0.4954 (0.2858–0.8587)*	*0.017*	*0.6209 (0.3564–1.082*	*0.0925*
Lymph node metastasis	0.4954 (0.2858–0.8587)	0.2723	–	–
Only lymph node metastasis	*0.4482 (0.2363–0.8502)*	*0.0145*	–	–
Brain metastasis	0.9460 (0.4728–1.8927)	0.876	–	–
Malignant pleural effusion		0.991	–	–
Bone metastasis	1.4029 (0.8022–2.4535)	0.2375	–	–
Adrenal gland metastasis	1.1271 (0.5095–2.4933)	0.7689	–	–
N. of metastatic sites	1.1610 (0.7966–1.6921)	0.4395	–	–
ECOG PS	*2.706 (1.774–4.126)*	*<* *0.0001*	*2.588 (1.655–4.046)*	*<* *0.0001*
Line of treatment	1.1507 (0.7571–1.7488)	0.5133	–	–
Global				*<* *0.0001*

Italic values refer to statistically significant covariates (p value < 0.05) for OS

**Table 3:** Univariate and multivariate analysis for PFS

Covariate	Univariate analysis		Multivariate analysis	
	HR (95% CI)	P value	HR (95% CI)	P value
Age (≥ 69 years)	0.7437 (0.4732–1.1688)	0.2015	–	–
Sex	1.313 (0.8109 -2.126)	0.268	–	–
Weight (≥ 69.5 kg)	1.1089 (0.6850–1.7950)	0.6756	–	–
Height (> 168 cm)	1.0502 (0.6274–1.7577)	0.8530	–	–
Liver metastasis	*2.9391 (1.6952–5.0957)*	*0.0001*	*3.456 (2.002–5.965)*	*< 0.0001*
Lung metastasis	*0.6133 (0.3818–0.9852)*	*0.0443*	–	–
Lymph node metastasis	0.7610 (0.4697–1.2330)	0.2697	–	–
Only Lymph node metastasis	*0.4324 (0.2567–0.7282)*	*0.0017*	–	–
Brain metastasis	1.0419 (0.5639–1.9251)	0.8964	–	–
Malignant pleural effusion	1.0225 (0.4452–2.3485)	0.9583	–	–
Bone metastasis	1.5459 (0.9538–2.5057)	0.0786	–	–
Adrenal gland metastasis	1.2286 (0.6145–2.4563)	0.5623	–	–
N. of metastatic sites	1.1524 (0.8550–1.5533)	0.3542	–	–
ECOG PS	*2.26 (1.602–3.187)*	< 0.0001	*2.260 (1.580–3.233)*	*<* *0.0001*
Line of treatment	1.0253 (0.7140–1.4724)	0.8928	–	–
Global				*<* *0.0001*

Italic values refer to statistically significant covariates (p value < 0.05) for PFS

To use the nomogram, a vertical line needs to be delineated to the point raw to assign point values for each variable. Thereafter, the corresponding points are to be summed to obtain the total points. Finally, from the total points a vertical line needs to be drawn to get the value of 3, 6, 12 months OS probability. The presence of lung metastases corresponds to 20 points, the presence of liver metastases corresponds to 0 points, while the ECOG PS of 1 corresponds to 50 points. The total point of 70 corresponds a 3- and 6-month OS of about 0.6 (60%) and 0.3 (30%), respectively.

**Fig. 4:**
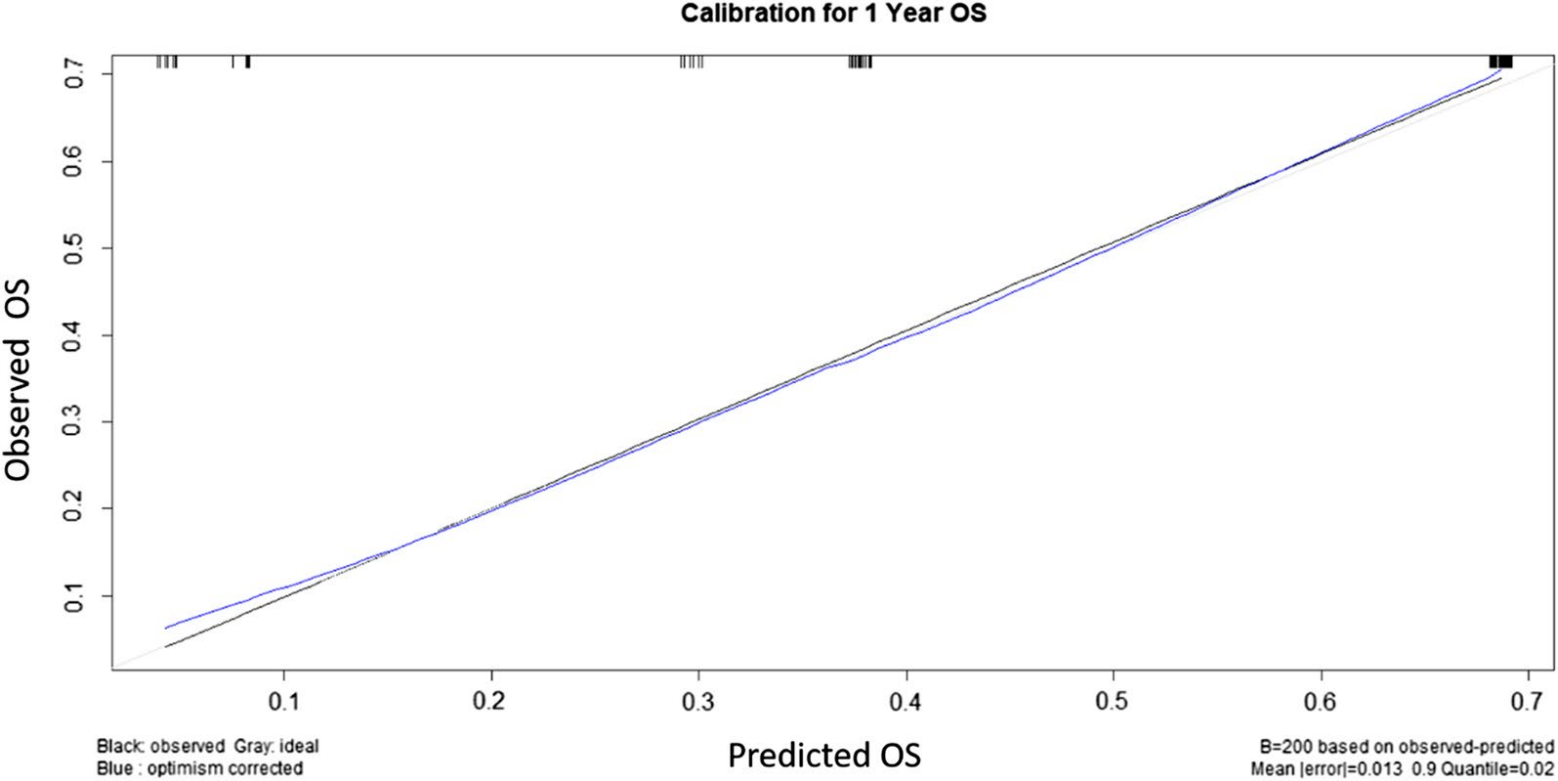
Calibration plot of the final nomogram for OS. All patients were grouped based on their predicted probabilities. Mean predicted probabilities were plotted against the actual incidence of PFS. The reference line represents perfect quality of observed frequencies and predicted probabilities

**Fig. 5:**
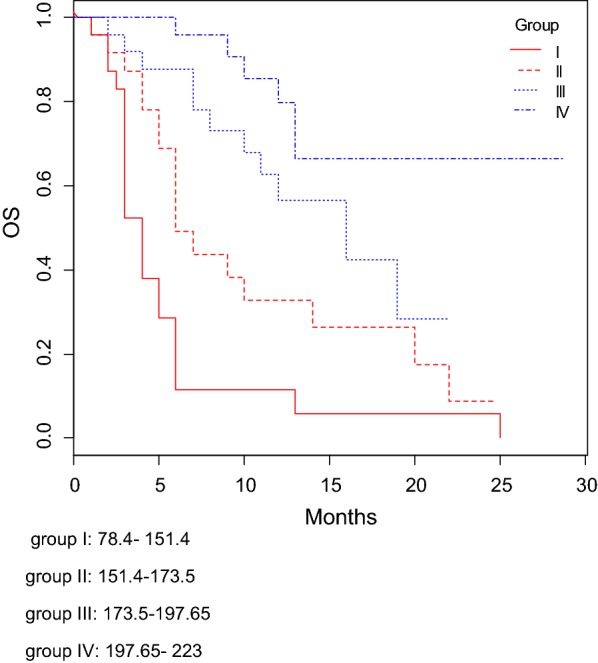
OS according to the nomogram total points (I–IV ranges). The groups were obtained considering the total point distribution of our population. Group I and II (red lines) represent patients with poor outcome

The C-indexes for OS models was 0.76 and calibration of the nomogram for OS was considered adequate (Fig. 4). Kaplan–Meier curves according to the range of total points highlighted the appropriateness of distinguish the patients’ survival in all the subgroups (Fig. 5). The groups were obtained considering the total point distribution of our population. Group I and II (red lines) represent patients with poor outcome.

The PFS was significantly shorter in patients with liver metastases (*P *< 0.0001) and higher ECOG PS score (*P *< 0.0001), while was longer in patients having only lung lymph nodes (*P *= 0.0017) and lung metastases (*P *= 0.0443). ECOG PS (*P *< 0.001) and liver metastases (*P *< 0.001) resulted statistically significant at MVA. The C-indexes for PFS models was 0.72.

## Discussion

In the last few years, an unprecedented number of immunotherapeutics including the anti-PD1 nivolumab have proven effective compared to standard chemotherapy in pretreated NSCLC patients that has greatly expanded treatment options beyond first-line. However, in spite of a proportion of them deriving a long-term disease control, roughly 60–80% of patients progress on ICI carrying a dismal prognosis [18]. Hence, the question on how to properly select the best candidates to second-line immunotherapy has rapidly risen and represents thus an urgent unmet need.

Of interest, some reports have proposed clinical prognostic factors in advanced-stage NSCLC treated with second-line nivolumab, among which is ECOG PS.

Our findings concerning the impact of patients’ general health conditions on nivolumab efficacy are aligned with those previously described in the literature. Indeed, poor ECOG PS (≥ 2) has been consistently shown to be an independent predictor of poorer survival in both clinical trials and real-life experience of nivolumab-treated patients [19–21]. With regards to the role of age, at the UVA, we found that elderly patients (> 70 years) were less likely than younger to benefit from nivolumab. Limited and conflicting data are available regarding the safety and efficacy of nivolumab in older people due to their underrepresentation in clinical trials and the lack of randomized studies in this specific subset of patients.

Among disease characteristics in our cohort, lung and lymph nodes metastases seemed to be favourable prognostic factors, while liver involvement emerged as a negative feature. Accordingly, a growing number of studies have been suggesting that ICI treatment may have a differential outcome based on the type of metastatic involvement. In particular, liver metastases have been independently associated with poorer survival in the real-world setting [16, 17]. Likewise, the updated follow-up of CheckMate 017 and CheckMate 057 demonstrated in the experimental arm 3-year OS of 8% vs. 17% in patients with liver metastases compared to the whole population [18]. Consistently, a cohort from Keynote 001 evaluating another anti-PD1 pembrolizumab confirmed a reduced response rate (28.6% vs. 56.7%) and shortened PFS in NSCLC patients with liver metastasis (mPFS 1.8 vs. 4 months, P = 0.0094), compared to those without liver metastasis [22]. To this end, accumulating preclinical evidence is shedding light on the differential response and clinical benefit seen with anti-PD1 agents according to metastatic sites and specifically on the poorer prognosis of patients with liver metastases.

It was demonstrated that liver metastases seem to be “colder” than a primary tumour or lung and lymph node metastases [23]. In addition, in the liver microenvironment, T cells have been shown to interact with sinusoidal endothelial cells, resulting in differentiation of the T cells into a Treg phenotype and/or in partial activation of the T cells, followed by passive cell death [24]. More interestingly, circulating levels of Eotaxin-2 and IP-10 that are chemokines attracting immunosuppressive immune cells have been reported to be higher in patients with liver metastatic involvement from both melanoma and colorectal cancer, further pointing toward unique immunosuppressive mechanisms sustained by liver metastases.

In the present study, we investigated the role of clinical features in order to build a nomogram enabling individualized OS estimation in a real-world cohort of advanced NSCLC receiving the anti-PD1 agent nivolumab as second- or later line of treatment. The prognostic nomogram is based on readily available, inexpensive and easily-to-collect patient (ECOG PS) and disease variables (liver and lung metastases).

The present study has some limitations to be acknowledged. This is a retrospective bicentric cohort study with a 11-month follow up and a relatively small sample size, thereby with potential for inherent biases. Still, the lack of data regarding PD-L1 expression determined by using the Tumor Proportion Score is another shortcoming of the analysis. Finally, external prospective validation is required to assess reproducibility and generalizability of our results.

## Conclusion

We developed an easy-to-use and inexpensive device to assist the clinician with a quantitative tool to predict OS probability in NSCLC treated with nivolumab in the clinical practice. The nomogram was built on the basis of clinico-pathological variables which retained independent prognostic value in the MVA and showed an adequate performance. While waiting for novel biomarkers, this prognostic tool could be valuable to more accurately driving treatment decision in daily practice and enrollment onto clinical trials.
